# Monitoring PfMDR1 transport in *Plasmodium falciparum*

**DOI:** 10.1186/s12936-015-0791-3

**Published:** 2015-07-15

**Authors:** Sarah J Reiling, Petra Rohrbach

**Affiliations:** Institute of Parasitology, McGill University, Ste. Anne de Bellevue (Montreal), QC H9X-3V9 Canada

**Keywords:** *Plasmodium falciparum*, Malaria, Fluo-4, Anti-malarial drugs, Chloroquine, Mefloquine, Quinine, Digestive vacuole, Transport

## Abstract

**Background:**

The *Plasmodium falciparum* multidrug resistance 1 transporter, PfMDR1, contains five amino acid polymorphisms that are suggested to be involved in altered drug transport from the parasite’s cytosol into the digestive vacuole (DV). Transport of a substrate into another intracellular compartment influences drug availability at its site of action, therefore making the parasite more susceptible or resistant to a drug. Fluo-4 is a known fluorescent substrate that can be used as a molecular tool to investigate transport dynamics of PfMDR1 in many parasite strains.

**Methods:**

Six *P. falciparum* strains with varying PfMDR1 mutations were loaded with Fluo-4 AM. Accumulation of the fluorophore in the DV was measured using confocal microscopy. The role of a key amino acid mutation was verified using selected parasite clones with point mutations at PfMDR1 amino acid position 1042. Equal expression of PfMDR1 was confirmed by Western blot.

**Results:**

Fluo-4 was transported by PfMDR1 into the DV of most drug-sensitive and -resistant parasites. Asparagine at PfMDR1 amino acid position 1042 was crucial for Fluo-4 transport, while the N1042D substitution abolished Fluo-4 transport. Competition studies of Fluo-4 with chloroquine, quinine and mefloquine were performed on parasites harbouring asparagine at position 1042. A distinct Fluo-4 transport inhibition pattern for each tested anti-malarial drug was observed in parasite strains of different genetic background.

**Conclusion:**

This study demonstrates that Fluo-4 can be used to investigate PfMDR1 transport dynamics in both drug-sensitive and -resistant parasites. Furthermore, direct evidence of altered Fluo-4 transport in PfMDR1 is linked to a single amino acid mutation in the substrate binding pocket. This system offers a great tool to investigate the role of substrate transport by PfMDR1 and the mutations necessary to support transport, which would lead to new insights for the development of novel anti-malarial drugs.

**Electronic supplementary material:**

The online version of this article (doi:10.1186/s12936-015-0791-3) contains supplementary material, which is available to authorized users.

## Background

Emerging resistance to commonly used anti-malarial drugs is a major setback in the fight against malaria worldwide [1]. Understanding the molecular mechanisms behind drug resistance is of high importance in ongoing efforts to control this disease.

Early on, researchers found a correlation between anti-malarial resistance and the *Plasmodium falciparum* multidrug resistance 1 transporter (PfMDR1) [2, 3]. PfMDR1 is a P-glycoprotein homologue (Pgh1) and belongs to the ATP binding cassette (ABC) transporter superfamily. It is a 162 kDa protein with two nucleotide binding domains (NBD) and twelve transmembrane domains (TMDs), with a putative substrate binding pocket in TMD11 [4]. The transporter is located in the membrane of the digestive vacuole (DV) [5], transporting substrates from the parasite’s cytoplasm into the DV [6].

Two factors have been suggested to play a role in altered drug susceptibility to specific anti-malarial drugs: PfMDR1 polymorphisms and *pfmdr1* gene duplications. Resistance has been associated with one or more variations at five amino acid positions in the PfMDR1 transporter, with wild-type PfMDR1 containing the amino acids N86, Y184, S1034, N1042, D1246. Increased *pfmdr1* copy numbers have been linked to mefloquine (MQ), lumefantrine (LF), halofantrine (HF), quinine (QN) and artemisinin (AS) resistance [7–9], while PfMDR1 amino acid mutations S1034C, N1042D, D1246Y were found to enhance parasite susceptibility to MQ, HF and AS, independent of gene copy number [2, 10].

Additional evidence for altered drug transport in wild-type versus mutant PfMDR1 was shown through expression of different *pfmdr1* variants in *Xenopus laevis* oocytes. Wild-type PfMDR1 transported QN and chloroquine (CQ) but not HF, while mutant PfMDR1 transported HF but not QN or CQ [11]. Furthermore, residue 184 altered transport kinetics independent of drug binding specificity [12].

Computational models of PfMDR1 describe a substrate-binding pocket that includes the amino acid residues 1034 and 1042 [4, 13]. The binding of several anti-malarial drugs was investigated using docking simulations within the PfMDR1 substrate-binding pocket. Among the tested drugs, MQ was the only candidate whose ability to form an H-bond with residue 1042 was completely abolished through the N1042D substitution [13].

Apart from anti-malarial drug transport, it was shown that several PfMDR1 drug-resistant variants could transport the fluorescent substrate Fluo-4 into the DV of the parasite [6]. In contrast, Fluo-4 transport was abolished in drug-sensitive HB3 parasites harbouring the PfMDR1 variant N86, F184, S1034, D1042, D1246. In a follow-up paper, the pump rate of PfMDR1 (F/Y86, Y184, S1034, N1042, D1246) was determined for Dd2 parasites using live cell imaging of intact infected erythrocytes [14].

In this study, drug-sensitive and -resistant *P. falciparum* strains of different genetic background and varying PfMDR1 polymorphisms were used to investigate the crucial mutations required for Fluo-4 transport. In addition, *P. falciparum* clones harbouring a single mutation at position 1042 were analysed. Aspartic acid at position 1042 was found to abolish Fluo-4 transport into the DV. This could be restored by replacing aspartic acid at position 1042 with asparagine. Furthermore, the N1042D substitution resulted in increased sensitivity to MQ. Using Fluo-4 as a competitive substrate offers a powerful tool to investigate the role of PfMDR1 in transport of currently used anti-malarial drugs for both drug-sensitive and -resistant parasites.

## Methods

### Parasite strains and culture conditions

Three CQ-sensitive (CQS) (3D7, D10, HB3) and three CQ-resistant (CQR) (Dd2, FCR3, FCB) *P. falciparum* strains, as well as stably transfected *P. falciparum* clones, derived from the parental lines GC03 and 3BA6, were used in this study. The two strains GC03 and 3BA6 are progeny of the HB3 × Dd2 genetic cross [15] and harbour the PfMDR1 variant from HB3 but differ in their PfCRT phenotype and genotype (Table 1). PfMDR1 mutants derived from GC03 and 3BA6 were produced by Sidhu and colleagues through partial *pfmdr1* gene replacement that substituted aspartic acid at position 1042 with asparagine while leaving amino acids at residues 1034 and 1246 unchanged. The resulting clones were: SND^GC03^ (S1034, N1042, D1246 in a GC03 genetic background), SND^3BA6^ (S1034, N1042, D1246 in a 3BA6 genetic background), as well as the recombinant controls SDD^GC03^ and SDD^3BA6^ [10]. All strains were cultured continuously, as described by Trager and Jensen [16], with modifications. Briefly, parasites were propagated at 5% haematocrit in culture medium containing RPMI 1640 (Life Technologies, Burlington, ON, Canada) supplemented with 25 mM HEPES, 2 mM l-glutamine, gentamicin (20 µg/ml) (Life Technologies, Burlington, ON, Canada), 100 µM hypoxanthine (Sigma-Aldrich, Oakville, ON, Canada), 0.5% AlbuMAX I (Life Technologies, Burlington, ON, Canada). Parasites were maintained at 37°C with an atmosphere of 5% CO_2_, 3% O_2_ and 92% N_2_. A^+^ red blood cells were obtained from the Interstate Blood Bank (Memphis, TN, USA). Giemsa-stained blood smears were prepared daily to monitor parasite growth. For synchronization, parasites were treated with 5% d-sorbitol (BioShop Canada, Burlington, ON, Canada) for 10 min at 37°C; sorbitol was removed and parasites were washed once before putting them back into culture. To obtain highly synchronous parasite cultures, sorbitol treatment was repeated after 6–8 h.

**Table 1:** Main PfCRT and PfMDR1 mutations of *Plasmodium falciparum* parasites used in this study

	PfCRT	PfMDR1				
	76	86	184	1034	1042	1246
**Strains**						
HB3	K	N	F	S	D	D
3D7	K	N	Y	S	N	D
D10	K	N	Y	S	N	D
Dd2	T	Y/F	Y	S	N	D
FCR3	T	Y	Y	S	N	D
FCB	T	Y	Y	S	N	D
**Clones**						
GC03	K	N	F	S	D	D
SDD^GC03^	K	N	F	S	D	D
SND^GC03^	K	N	F	S	N	D
3BA6	T	N	F	S	D	D
SDD^3BA6^	T	N	F	S	D	D
SND^3BA6^	T	N	F	S	N	D

### DNA isolation and sequence analysis

The full-length sequence of *pfmdr1* and partial sequence of *pfcrt* was verified for all strains. Parasite strains were grown to ≥5% parasitaemia and DNA was extracted using the QIAamp DNA Blood Mini Kit (Qiagen, Toronto, ON, Canada) according to manufacturer’s instructions. DNA was amplified in overlapping PCR fractions using HotStarTaq DNA polymerase (Qiagen, Toronto, ON, Canada). To account for the AT-rich nucleotide content in the *P. falciparum* genome, dNTPs (Invitrogen Canada, Burlington, ON, Canada) were mixed at 75% AT and 25% GC. For PCR optimization, 2 mM MgCl_2_, 300 µM dNTPs and 300 nM primers were used for the reaction. For each reaction mix, 20 ng genomic DNA was used. PCR reactions consisted of an initial activation step of 94°C for 3 min, followed by 35 cycles of 94°C for 60 s, 49–61°C (adjusted for each primer pair) for 30 s, and 72°C for 1 min. Primers used for *pfmdr1* gene sequencing are described in [14]. Primers used for sequencing the *pfcrt* gene region containing the main mutation sites were: *pfcrt_F*: 5′-GGAGGTTCTTGTCTTGGTAAATG, *pfcrt_R*: 5′-TGGTAGGTGGAATAGATTCTCTTATAAA. Samples were sent for sequencing to Genome Quebec, Canada and analysed using the BioEdit software [17].

### Protein expression

To determine PfMDR1 protein levels, 20 µg whole cell protein lysates were loaded in each lane of an 8% acrylamide gel containing SDS. The proteins were transferred onto a PVDF membrane, which was then blocked O/N at 4°C with 5% milk (w/v) and 0.05% Tween-20 (ACP Chemicals, St-Leonard, QC, Canada) in phosphate buffered saline (PBS). The membrane was further incubated with the appropriate dilution of primary anti-PfMDR1 (kindly provided by Prof Cowman, Walter and Eliza Hall Institute, VIC, Australia) or anti-PfHSP70 (GenWay Biotech, San Diego, CA, USA) antibody (1:2,000) in PBS-T + 5% milk for 1 h (RT), then washed and incubated with HRP-conjugated anti-rabbit IgG secondary antibody (Abcam, Toronto, ON, Canada) (1:20,000) for 1 h (RT). Immunoreactive bands were detected with ImmunStar WesternC Chemiluminescent Kit (Bio-Rad Laboratories, Mississauga, ON, Canada) using a myECL Imager (Thermo Scientific, Burlington, ON, Canada). To confirm equal protein loading, chemiluminescence intensities of PfMDR1 were calculated for each parasite clone relative to the respective PfHSP70 chemiluminescence using ImageJ 1.47q (National Institutes of Health, USA).

### Growth inhibition assay

Growth inhibition assays were performed as described previously [18], with modifications. Briefly, synchronized ring stage parasites were diluted to a final parasitaemia of 0.5% and a haematocrit of 2%. A total of 100 µl culture medium per well was prepared in a 96-well plate assay, with a drug dilution series of 1:3, ranging from 1 µM to 0.15 nM. Plates were incubated at 37°C, 5% CO_2_, 3% O_2_ and 92% N_2_ for 72 h, then frozen and stored at −80°C. Plates were thawed at room temperature and 100 µl 2× lysis buffer (20 mM Tris pH 7.5, 5 mM EDTA, 0.008% saponin, 0.08% Triton X-100, 0.2 µl SYBR Green I/ml) was added to each well. Plates were incubated in the dark for at least 1 h. Fluorescence intensity was determined using a Synergy H4 plate reader (Fisher Scientific, Nepean, ON, Canada) with 485 nm excitation and 520 nm emission wavelengths. IC_50_ values were determined by fitting concentration response curves with a custom-made procedure for IGOR Pro 6.2 based on an R script kindly provided by Le Nagard [19, 20].

### Live cell imaging

Synchronized trophozoite stage parasites were loaded with 5 µM Fluo-4 AM (Life Technologies, Burlington, ON, Canada) in Ringer’s solution (122.5 mM NaCl, 5.4 mM KCl, 1.2 mM CaCl_2_, 0.8 mM MgCl_2_, 11 mM d-glucose, 10 mM HEPES, 1 mM NaH_2_PO_4_, pH 7.4) for 50 min at 37°C. Cells were then washed twice with Ringer’s solution and transferred to a microscope chamber. Parasites were kept at 37°C during microscopy using a stage-top incubator (Tokai Hit, Shizuoka-ken, Japan). A series of four images per parasite was taken using a Zeiss LSM710 confocal microscope (Carl Zeiss, Oberkochen, Germany) equipped with a water-corrected objective (C-apochromat 63×/1.20 W Korr M27) and a 488 nm laser (12.5 mW, 2% intensity). The range of emitted fluorescence was measured from 493 to 622 nm. Images were analysed using ImageJ 1.47q (National Institutes of Health, USA).

## Results and discussion

### PfMDR1 transports Fluo-4 into the digestive vacuole

A genetic linkage between Fluo-4 accumulation in the DV and the PfMDR1 transporter was previously described [6]. Although variations in Fluo-4 accumulation were observed between parasite strains harbouring different PfMDR1 mutations, the amino acid mutation(s) responsible for Fluo-4 transport remained to be determined. To identify PfMDR1 mutation(s) crucial for Fluo-4 transport, several drug-sensitive and -resistant *P. falciparum* strains of different genetic backgrounds were tested for accumulation of Fluo-4 in the DV. Three CQS and three CQR strains harbouring different PfMDR1 mutations were selected for these experiments (Table 1). While PfMDR1 has been suggested to play a role in CQ resistance, the key genetic indicator for CQS versus CQR parasites is attributed to the amino acid mutation K76T in the *P. falciparum* chloroquine resistance transporter (PfCRT) [21]. This mutation was taken into consideration in this study and the relevance of PfMDR1 polymorphisms is discussed for strains harbouring either lysine (K76) or threonine (K76T) in PfCRT.

**Figure 1:**
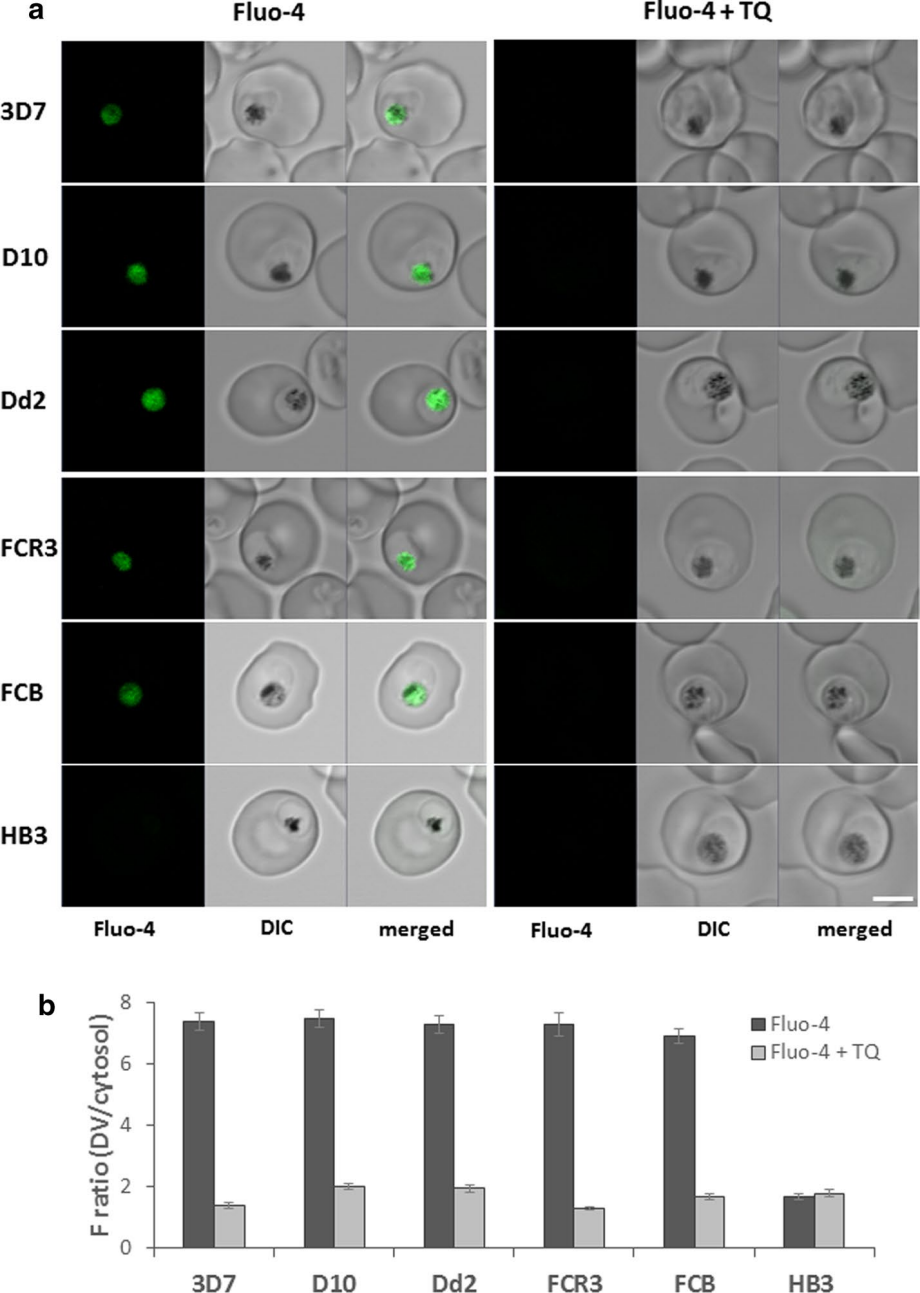
Fluo-4 fluorescence in *P. falciparum* parasites. Parasites were incubated with 5 µM Fluo-4. **a** Representative images of *P. falciparum*-infected erythrocytes. *Scale bar* 5 µm. **b** Mean Fluo-4 fluorescence ratio (DV/cytosol) ± SEM. When treated with the Pgp inhibitor tariquidar (TQ), fluorescence ratio was reduced, indicating that Fluo-4 transport occurred exclusively through PfMDR1. Total n ≥ 67 for each strain, done in three independent experiments on different days.

Of the five mutation sites in PfMDR1 known to play a role in drug resistance, the parasite strains used for these experiments contained mutations at amino acid positions 86, 184 and 1042, which are mainly found in African and Asian isolates [7, 22–24]. To determine if mutations at these residues influence transport of Fluo-4 via PfMDR1, accumulation of Fluo-4 in the DV was measured in live parasites using confocal microscopy. All parasite strains, except HB3, showed high accumulation of Fluo-4 in the DV (Figure 1a, b). Fluo-4 accumulation in the DV was impeded by pre-incubation of the parasites with tariquidar (TQ) (Figure 1b), a specific inhibitor of Pgp transporters [25]. Importantly, Fluo-4 accumulation in the DV was shown to be independent of the PfCRT K76T mutation, suggesting that the PfCRT mutation alone has no effect Fluo-4 accumulation.

### Fluo-4 transport is associated with PfMDR1 N1042

The parasite strain HB3, which does not accumulate Fluo-4 in the DV, harbours two PfMDR1 polymorphisms—Y184F and N1042D—not found in the other tested *P. falciparum* strains (Table 1), suggesting that one of these two residues is responsible for the altered Fluo-4 transport seen in HB3 parasites. It has previously been demonstrated for the human homolog of PfMDR1 that the Y184F mutation does not influence drug specificity [12]. Furthermore, this residue only mildly increased drug resistance in comparison to other PfMDR1 mutation sites [4]. In contrast, amino acid position 1042 is thought to be part of the transporter binding pocket and forms electrostatic interactions with amodiaquine, CQ, MQ (only in the presence of asparagine), HF, vinblastine and vincristine [4, 10, 13]. Therefore, the role of PfMDR1 mutations at position 1042 was investigated in more detail.

**Figure 2:**
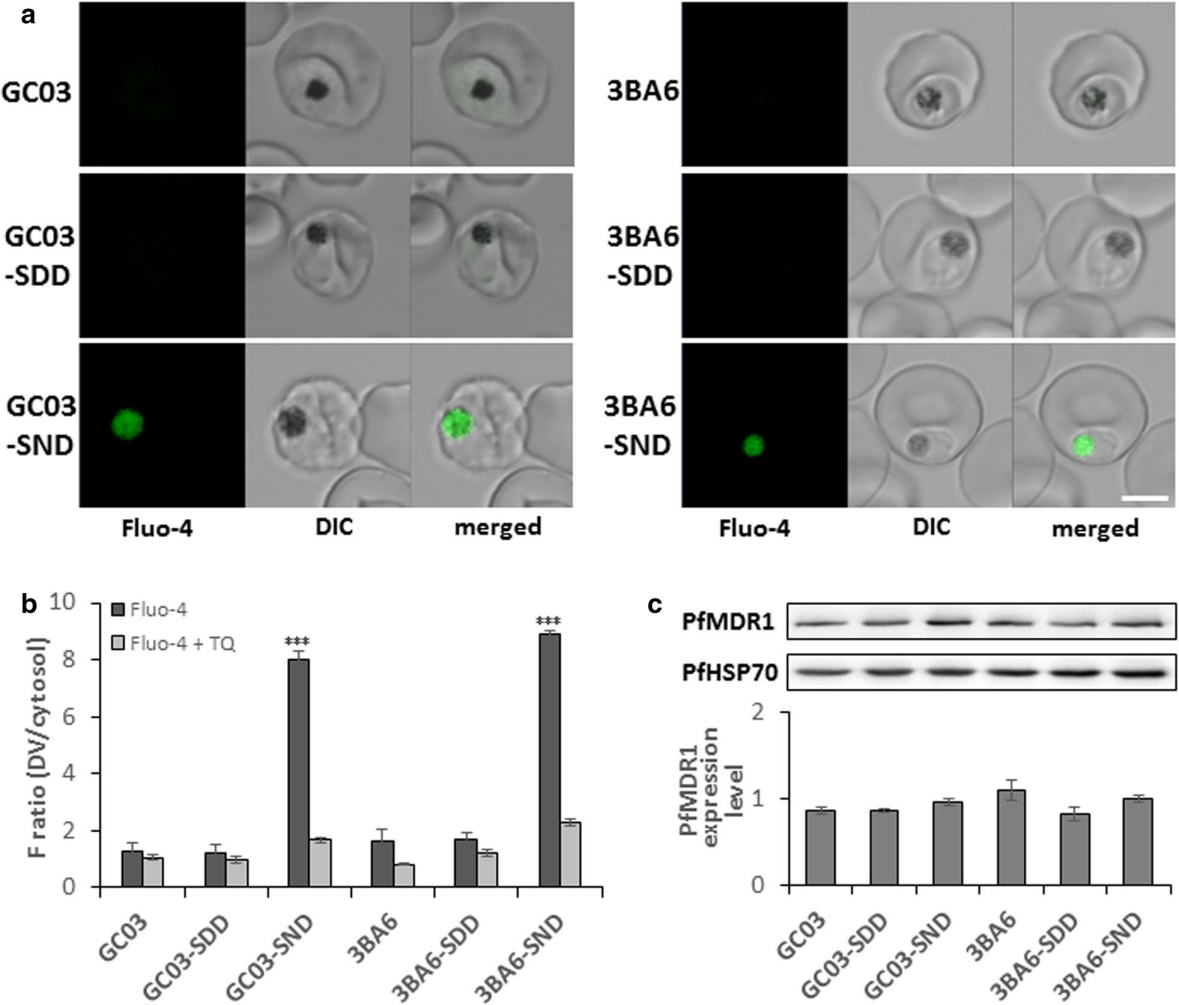
Fluo-4 fluorescence in *P. falciparum* clones with varying PfMDR1 and PfCRT mutations. Parasites were incubated with 5 µM Fluo-4 AM in Ringer’s solution for 50 min at 37°C, then washed with Ringer’s solution and transferred onto a microscope chamber. **a** Single images of *P. falciparum* clones. *Scale bar* 5 µm. **b** Mean Fluo-4 fluorescence ratio measured from the digestive vacuole (DV) and the cytosol. When treated with tariquidar (TQ), parasites were pre-incubated with 100 nM TQ for 10 min at 37°C before adding Fluo-4 AM for 50 min. Total n ≥ 80 for each strain, done in three independent experiments on different days. *Error bars* represent SEM. ***p < 0.0001. **c** Western blot of synchronized trophozoite stage parasites using anti-PfMDR1 antibodies. Anti-PfHSP70 was used as a loading control. PfMDR1 protein expression levels were normalized to PfHSP70 and measured in triplicate ± SEM.

To verify the influence of PfCRT on Fluo-4 transport, existing parasite clones containing either lysine or threonine at residue 76 in PfCRT were selected and compared. For this, stably transfected *P. falciparum* clones derived from the parental lines GC03 and 3BA6 were used, where GC03 harbours the K76 PfCRT genotype and is CQ sensitive, and 3BA6 contains the PfCRT K76T mutation that confers CQ resistance (Table 1). Both GC03 and 3BA6 contain the PfMDR1 sequence N86, F184, S1034, D1042, D1246, identical to the HB3 strain. Experiments verified that neither GC03 nor 3BA6 were able to accumulate Fluo-4 in the DV, as expected (Figure 2a, b).

As a control, parasite clones SDD^GC03^ and SDD^3BA6^, harbouring the same mutation as GC03 and 3BA6, revealed that the PfMDR1 N1042D mutation did not transport Fluo-4 into the DV (Figure 2a, b). Fluo-4 transport was only established with the introduction of asparagine at residue 1042 for both clones (SND^GC03^ and SND^3BA6^). Transport was independent of the PfCRT K76T mutation, since Fluo-4 transport was equally impaired in GC03 and 3BA6 parental lines as well as the SDD^GC03^ and SDD^3BA6^ controls, and only seen in the clones SND^GC03^ and SND^3BA6^ (Figure 2a, b). Transport specificity for PfMDR1 in clones SND^GC03^ and SND^3BA6^ was confirmed through pre-incubation of the samples with the Pgp inhibitor TQ (Figure 2b; Additional file 1: Figure S1). These results indicate a pivotal role for asparagine at PfMDR1 residue 1042 in Fluo-4 transport, independent of PfCRT.

In addition to PfMDR1 polymorphisms, increased *pfmdr1* gene copy number [8, 9, 26] and protein expression levels have also been suggested to play a role in drug resistance or susceptibility. To verify that Fluo-4 accumulation in the DV of clones SND^GC03^ and SND^3BA6^ was not linked to increased PfMDR1 expression, PfMDR1 protein levels were analysed for these parasite clones (Figure 2c). No significant increase in PfMDR1 expression was detected for the GC03 and 3BA6 parental lines or their clones harbouring the D1042N substitution (p > 0.05). Therefore, Fluo-4 accumulation in the DV of these clones was only associated with the PfMDR1 mutation at residue 1042.

### Effect of N1042D polymorphism on drug susceptibility

Since transport of Fluo-4 was dependent on specific PfMDR1 polymorphisms, it follows that the N1042D substitution in the PfMDR1 substrate-binding pocket may not only alter the binding and transport of Fluo-4 but also influence other substrates, such as anti-malarial drugs. To determine if the amino acid substitution at residue 1042 resulted in altered drug sensitivity or resistance of the parasite clones, growth inhibition assays were performed. A change in a given drug’s IC_50_ would indicate a role for PfMDR1 amino acid position 1042 in its transport. CQ IC_50_ values for CQS clones were found to be 47 ± 2.6 nM for GC03, 46 ± 2.2 nM for SDD^GC03^ and 49 ± 2.8 nM for SND^GC03^, while CQ IC_50_ values for CQR clones were 262 ± 5.7 nM for 3BA6, 204 ± 9.4 nM for SDD^3BA6^ and 274 ± 5.6 nM for SND^3BA6^ (Table 2). This suggests that an amino acid mutation at PfMDR1 residue 1042 does not affect susceptibility or resistance to CQ. CQ resistance could be reversed in the CQR strains through the addition of 1 µM verapamil, while no significant difference was observed in CQS strains (p > 0.05), as expected. Similarly, CQS clones were more susceptible to quinacrine (QC) with IC_50_ values of 11 ± 0.3 nM for GC03, 13 ± 0.3 nM for SDD^GC03^ and 14 ± 0.4 nM for SND^GC03^ compared to the higher QC IC_50_ values of 43 ± 3.4 nM for 3BA6, 41 ± 1.0 nM for SDD^3BA6^ and 42 ± 2.3 nM for SND^3BA6^. Here again, as for CQ, an amino acid mutation at PfMDR1 residue 1042 did not affect susceptibility or resistance to QC. The same was found for dihydroartemisinin (DHA). CQS clones were fourfold more resistant than CQR clones (4 ± 0.1 vs 1 ± 0.1 nM) and DHA resistance was not reversible by the addition of 1 µM verapamil. Therefore, the PfMDR1 N1042D mutation alone does not appear to be a major determinant for CQ, QC or DHA resistance in these parasites (Table 2).

**Table 2:** IC_50_ values of *Plasmodium falciparum* parasites used in this study

	CQ	CQ + VP	DHA	DHA + VP	QC	QC + VP	QN	MQ
3D7	24 ± 5.6	17 ± 1.2	n.d.	n.d.	n.d.	n.d.	23 ± 1.8	8 ± 1.2
Dd2	169 ± 3.9	53 ± 6.5	n.d.	n.d.	n.d.	n.d.	283 ± 39.8	27 ± 4.0
GC03	47 ± 2.6	46 ± 2.1	4 ± 0.1	4 ± 0.1	11 ± 0.3	18 ± 0.4	85 ± 9.6	6 ± 0.9
SDD^GC03^	46 ± 2.2	47 ± 0.6	4 ± 0.1	3 ± 0.2	13 ± 0.3	15 ± 0.7	76 ± 4.9	7 ± 0.6
SND^GC03^	49 ± 2.8	47 ± 2.2	4 ± 0.1	3 ± 0.1	14 ± 0.4	17 ± 0.3	52 ± 3.5	19 ± 0.6
3BA6	262 ± 5.7	41 ± 1.8	1 ± 0.1	1 ± 0.1	43 ± 3.4	47 ± 2.2	83 ± 4.8	5 ± 0.3
SDD^3BA6^	204 ± 9.4	63 ± 1.5	1 ± 0.0	1 ± 0.1	41 ± 1.0	39 ± 0.5	78 ± 5.3	6 ± 0.7
SND^3BA6^	274 ± 5.6	69 ± 5.6	1 ± 0.1	1 ± 0.0	42 ± 2.3	39 ± 0.4	48 ± 2.4	14 ± 0.5

All values are given in nM. Experiments were done in triplicate on three separate days.

*CQ* chloroquine, *VP* verapamil, *DHA* dihydroartemisinin, *QC* quinacrine, *QN* quinine, *MQ* mefloquine, *n.d.* not determined.

Interestingly, the N1042D mutation did provide changes in drug susceptibility for the anti-malarial drugs QN and MQ. A significant decrease in QN IC_50_ values was observed in the GC03 and 3BA6-derived clones SND^GC03^ and SND^3BA6^ compared to the parental lines GC03 and 3BA6 or their recombinant controls SDD^GC03^ and SDD^3BA6^ (p < 0.005) (Table 2), which is in agreement with previous findings [10]. The opposite effect was observed for MQ. For the clones SND^GC03^ and SND^3BA6^clones, the D1042N substitution led to an approximate threefold increase in resistance to MQ. MQ IC_50_ values increased from 6 ± 0.9 nM in GC03 to 19 ± 0.6 nM in SND^GC03^, and from 5 ± 0.3 nM in 3BA6 to 14 ± 0.5 nM in SND^3BA6^. This was a significant increase in MQ IC_50_ values for the clones SND^GC03^ and SND^3BA6^ compared to the parental lines (p = 0.0003 for GC03 vs SND^GC03^; p = 0.0001 for 3BA6 vs SND^3BA6^). Similar increases in MQ resistance (approximately threefold) have been found in field isolates from Africa and Asia [27, 28]. A threefold increase in MQ resistance was also confirmed in vitro through allelic exchange of *pfmdr1* [2]. Therefore, the PfMDR1 mutation crucial for MQ resistance in vitro is likely to play a role in increased MQ resistance in the field. Nevertheless, growth inhibition experiments do not fully elucidate altered transport of MQ by PfMDR1. For this reason, co-incubation of anti-malarial drugs with Fluo-4 can provide additional insight into the role of PfMDR1 transport in drug resistance.

### Substrate competition of anti-malarial drugs with Fluo-4 to determine drug transport by PfMDR1

PfMDR1 is involved in the transport of substrates, including various anti-malarial drugs, from the cytosol into the DV [10, 11] but the role of PfMDR1 polymorphisms in drug resistance is not fully understood. Mutations at residue 86 has been suggested to allosterically influence the TMD11 drug binding site [4], while PfMDR1 residues 1034 and 1042 are located in a proposed binding pocket. Electrostatic interactions with both asparagine and aspartic acid at position 1042 have been demonstrated in silico for CQ, QN and MQ [4, 13]. While CQ was unable to form a hydrogen (H)-bond with either asparagine or aspartic acid at residue 1042, asparagine at residue 1042 was able to form a H-bond with MQ [4, 13]. No information is available on H-bond formation of QN with residue 1042.

Further insight into the importance of PfMDR1 in drug transport can be achieved through competition studies using two potentially competitive substrates, e.g., fixed amounts of Fluo-4 and increasing drug concentrations. For this purpose, two parasite strains were chosen to evaluate potential differences in anti-malarial drug transport in sensitive (3D7) and resistant (Dd2) parasites. 3D7 is sensitive to CQ, QN and MQ (Table 2), while Dd2 is resistant to these drugs. HB3 was not used, since it is not able to transport Fluo-4 into the DV. The measured changes of Fluo-4 accumulation in the DV in the presence of anti-malarial drugs were compared for 3D7 and Dd2 parasites.

**Figure 3:**
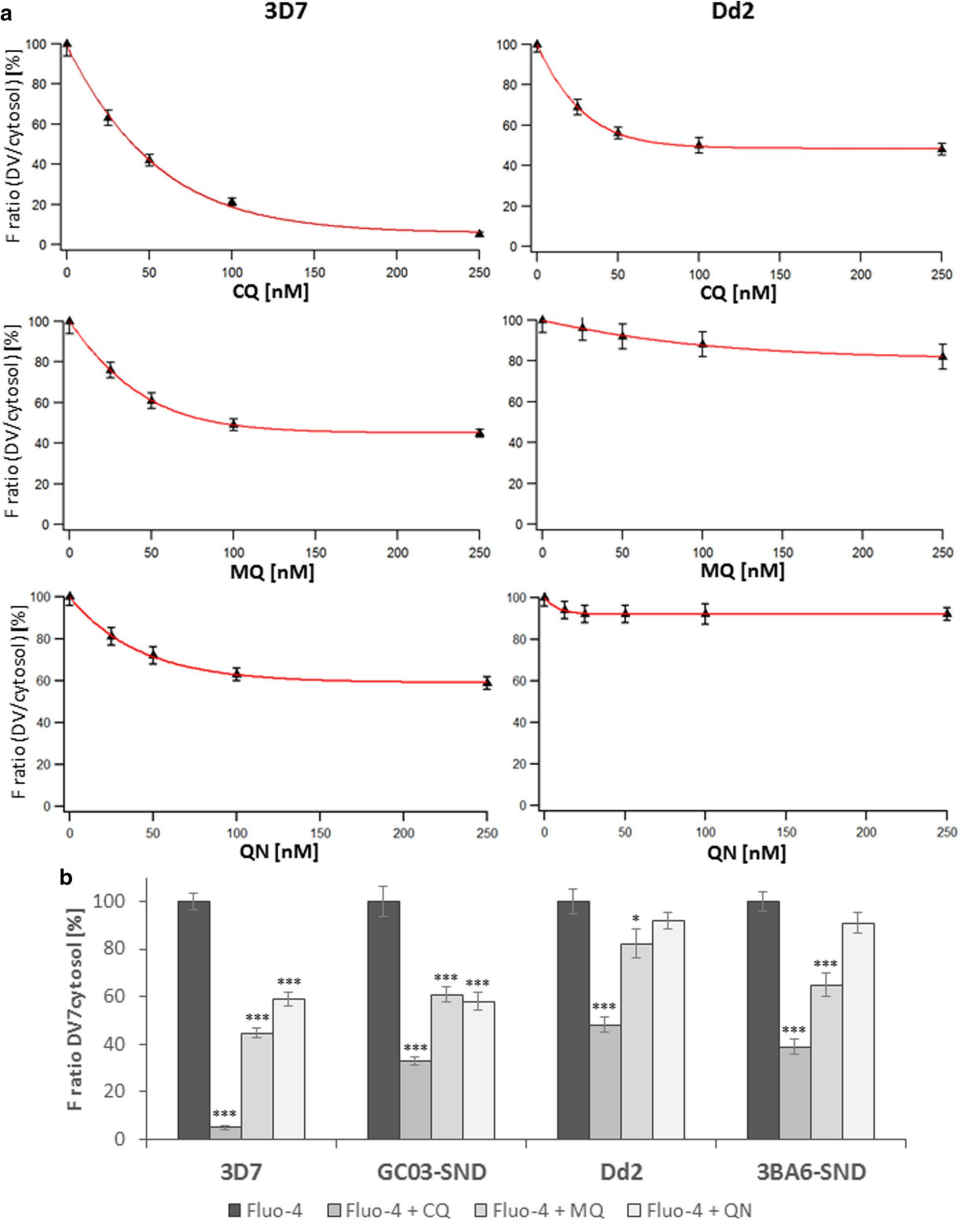
Competition of Fluo-4 transport with anti-malarial drugs. **a** Parasites were pre-incubated with different concentrations of chloroquine (CQ), mefloquine (MQ) or quinine (QN), or left untreated before adding Fluo-4 AM. **b** Parasites were pre-incubated as described with 250 nM of CQ, MQ or QN. Experiments were done in triplicate 3 independent days. *Error bars* represent SEM. *P < 0.05; ***P < 0.0001.

For 3D7 parasites, increasing CQ concentration led to the strong decrease in Fluo-4 accumulation in the DV (Figure 3a). While TQ is a non-competitive inhibitor of MDR1 through inhibition of the ATPase activity [25], CQ is thought to bind to the transporter’s substrate-binding pocket and is likely a competitive inhibitor of Fluo-4. Pre-incubation of 3D7 parasites with 250 nM CQ resulted in decreased Fluo-4 accumulation in the DV, where only 5 ± 1.0% Fluo-4 fluorescence was measured when compared to non drug-treated parasites. In Dd2 parasites, pre-incubation with 250 nM CQ reduced Fluo-4 fluorescence in the DV by half (48 ± 3.0%). MQ reduced Fluo-4 transport into the DV to 45 ± 2.0% in 3D7 parasites and only 82 ± 6.0% in Dd2 parasites (Figure 3a). For MQ, a decrease in Fluo-4 transport in 3D7 compared to Dd2 parasites cannot be attributed to a PfMDR1 amino acid mutation at position 1042 alone since both strains harbour wild-type N1042. However, Dd2 parasites harbour an amino acid mutation at PfMDR1 residue 86, which was described to influence MQ sensitivity in African field isolates [24, 30, 31]. This N86Y mutation may alter MQ transport by PfMDR1, as shown in the experiments presented here. QN was less effective in reducing Fluo-4 accumulation than CQ or MQ and only decreased Fluo-4 fluorescence in the DV to 59 ± 2.9% in 3D7 parasites and not significantly in Dd2 parasites (92 ± 3.4%) (Figure 3a). The differences between 3D7 and Dd2 parasites at the level of Fluo-4 reduction may be linked to PfMDR1 residue 86, which is suggested to influence CQ susceptibility [29]. It is conceivable that N86Y can allosterically influence the drug binding site at TMD11, as suggested in [4]. Residue 86 was not examined in this study.

Fluo-4 competition with anti-malarial drugs was also tested on the parasite clones SND^GC03^ and SND^3BA6^. Using a single drug concentration of 250 nM, Fluo-4 accumulation was decreased in the DV of SND^GC03^ and SND^3BA6^ parasites to 33 ± 1.9% and 39 ± 3.0% for CQ, 61 ± 3.1% and 65 ± 4.8% for MQ, and 58 ± 3.9% and 91 ± 4.4% for QN, respectively. Almost all tested anti-malarial drugs decreased Fluo-4 accumulation in the DV in both SND^GC03^ and SND^3BA6^ clones, except for QN (Figure 3b). This again suggests that the influence of anti-malarial drugs on substrate transport by PfMDR1 is not only dependent on PfMDR1 polymorphisms but also on the varying genetic background of the parasite strain.

**Figure 4:**
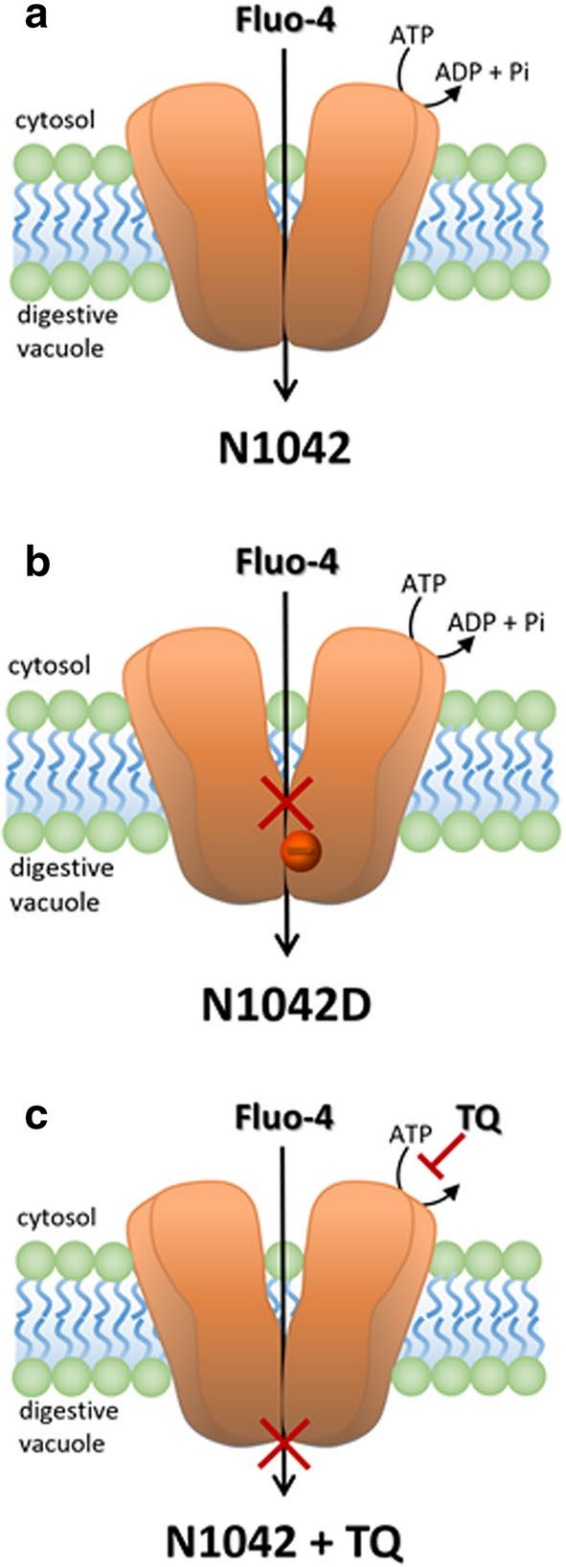
Model of Fluo-4 transport. The docking of substrates in the PfMDR1 binding pocket is influenced by the size, charge and polarity of local amino acids. Asparagine (N) and aspartic acid (D) are both polar, hydrophilic and small in volume. While N is an amide and uncharged, D is acidic and negatively charged. **a** For parasites containing the PfMDR1 N1042 polymorphism, the intrinsically negatively charged Fluo-4 gets transported from the cytoplasm into the digestive vacuole (DV) where it accumulates. **b** In the presence of PfMDR1 N1042D, which adds a negative charge to the binding pocket, Fluo-4 does not get transported by PfMDR1 and no Fluo-4 accumulation is detected in the DV. **c** Fluo-4 transport is abolished through the addition of the Pgp inhibitor tariquidar (TQ), which does not alter the substrate binding but prevents ATP hydrolysis and therefore the conformational change that is necessary for substrate dislocation.

The influence of the N1042D substitution on Fluo-4 transport is explained in a proposed model (Figure 4). Fluo-4 is negatively charged and replacement of a neutral asparagine at residue 1042 with a negatively charged aspartic acid alters the local environment, making it less favourable to Fluo-4 transport. TQ does not influence Fluo-4 affinity for the substrate-binding pocket but prevents adenosine triphosphate (ATP) hydrolysis of PfMDR1. Since both NBDs act in concert [32], complete inhibition of ATPase activity by TQ can be achieved when one nucleotide-binding site is blocked.

Detailed transport kinetics for parasite strains of different origin will provide additional information on substrate transport via PfMDR1. Using Fluo-4 as a competitive substrate circumvents the issue of direct labelling of anti-malarial drugs with a fluorescent tag that may alter their transport properties. Therefore, Fluo-4 is a powerful tool to selectively study transport kinetics of PfMDR1 in intact *P. falciparum*-infected erythrocytes.

## Conclusion

This study provides direct evidence for reduced PfMDR1-driven substrate transport through the single amino acid mutation N1042D, located in the substrate-binding pocket. The relevance of this amino acid mutation may have been underestimated and needs further investigation. Furthermore, it is now possible to test additional mutations within the binding pocket of PfMDR1 to verify their potential role in drug transport. Accordingly, using Fluo-4 in drug competition assays provides a powerful tool to better examine drug transport kinetics via PfMDR1. This newly acquired information can help elucidate the role of PfMDR1 in drug transport, which has remained controversial for decades. For example, while earlier investigations have suggested a link between MQ resistance and asparagine at PfMDR1 amino acid position 86 in isolates from Thailand and West Africa [30, 33], others have associated wild-type PfMDR1 and increased gene copy numbers with MQ resistance [3, 34]. More recent publications have found strong evidence for a role for N1042 in MQ resistance in Thai isolates [35, 36], suggesting that the importance of this mutation has been underestimated in previous field studies. The importance of N1042 for MQ resistance was similarly demonstrated in vitro through combined amino acid substitutions that generated a parasite strain harbouring the mutations S1024C, N1042D, D1246Y [2]. This newly generated strain was approximately threefold more susceptible to MQ exposure compared to the parental strain [2]. The results presented here support a potential role of PfMDR1 N1042 in parasite resistance to MQ. Further investigations will help elucidate the significance of this polymorphism on substrate transport.

## Additional file

Additional file 1:
**Figure S1.** Inhibition of Fluo-4 transport through tariquidar. *Plasmodium falciparum* clones were pre-incubated with 100 nM tariquidar for 10 min at 37°C, then 5 µM Fluo-4 AM was added for 50 min. Fluo-4 accumulation in the digestive vacuole was diminished. Experiments were done in triplicate on different days. These images are supplementary to the data obtained for Figure 2. Scale bar, 5 µm.
