# Supplementary figures and images for: Monitoring PfMDR1 transport in Plasmodium falciparum
Source: Malar J. 2015 Jul 15;14:270. doi: 10.1186/s12936-015-0791-3 (PMC4501111; doi:10.1186/s12936-015-0791-3)

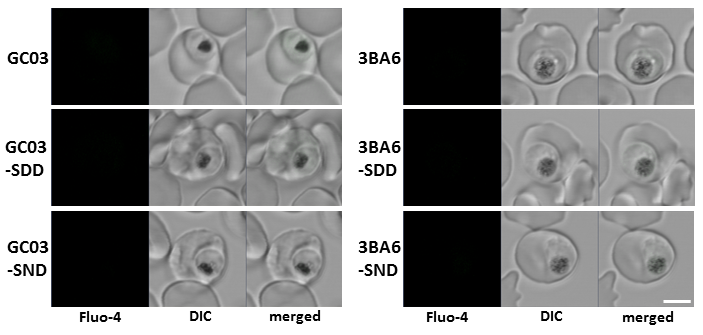

Supplement: Additional file 1: — Figure S1. Inhibition of Fluo-4 transport through tariquidar. Plasmodium falciparum clones were pre-incubated with 100 nM tariquidar for 10 min at 37°C, then 5 µM Fluo-4 AM was added for 50 min. Fluo-4 accumulation in the digestive vacuole was diminished. Experiments were done in triplicate on different days. These images are supplementary to the data obtained for Figure 2. Scale bar, 5 µm. [file 12936_2015_791_MOESM1_ESM.png]
